# A Survey of Demographics and Treatments in Melanoma Case Reports: Retrospective Bibliometric Analysis

**DOI:** 10.2196/56684

**Published:** 2024-04-22

**Authors:** Ross O'Hagan, Jessie Ngandjui, Benjamin Ungar, Jonathan Ungar, Nicholas Gulati

**Affiliations:** 1 Department of Dermatology Icahn School of Medicine at Mount Sinai New York, NY United States; 2 Department of Medical Education Garnet Health Middletown, NY United States

**Keywords:** melanoma, surgery, chemotherapy, immunotherapy, radiation therapy, case reports

## Abstract

Melanoma case reports show variations in treatment by age and sex.

## Introduction

Case reports provide valuable insights into clinical practices. However, dermatological case reports are not perfect, with some diseases being overreported and others having sex imbalances relative to patient populations [1]. Melanoma is a skin cancer that has differences in outcomes based on patient demographics [2,3]; thus, it is important to understand the treatments reported in case reports and their demographic variations. Therefore, we assessed the demographics represented in melanoma case reports, the various treatment modalities listed, and how treatments vary by demographics.

## Methods

To explore the demographics of patients in PubMed-listed case reports, we used techniques previously described [1,4]. Patients with melanoma and their treatment regimens were determined via string match. Included patients had the text “melanoma” listed in their case report summary. Each treatment modality was included in the analysis if its name was found in the case report summary. Age and sex information was listed in the PMC-Patients database. Differences in treatment by sex and mean age were determined by calculating odds ratios (ORs). Analysis was performed using R (version 4.2.2; R Foundation for Statistical Computing).

## Results

**Figure 1:**
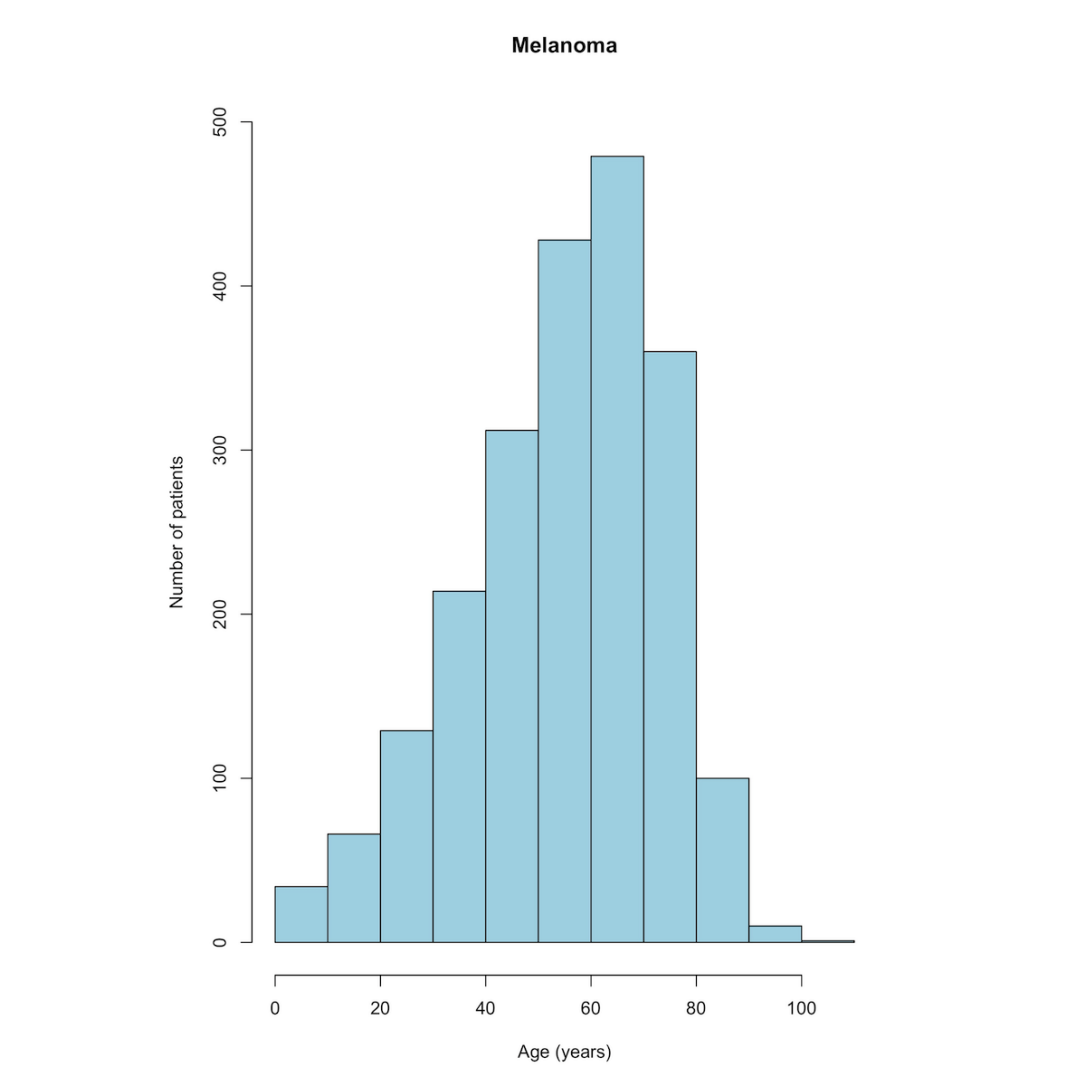
Age histogram of patients with melanoma.

Of the 167,034 patients listed in the PMC-Patients database, 2133 (1.3%) had case reports that mentioned “melanoma.” The mean age of patients with melanoma was 55.4 (SD 18.3) years (Figure 1), and 1173 (55%) of the 2133 patients were male.

Of the 2133 patients, the most mentioned treatment modality was surgery (n=693, 32.5% patients). The least frequently mentioned modality of treatment was radiation therapy (n=156, 7.3% patients; Table 1). Of the chemotherapies mentioned, the most common was dacarbazine (n=102, 4.8% patients). Of the immunotherapies mentioned, the most common was ipilimumab (n=341, 16% patients; Table 1).

Female patients were significantly more likely to receive surgery than male patients (OR 1.27, 95% CI 1.06-1.53; *P*=.009), and male patients were significantly more likely to receive immunotherapy (OR 1.34, 95% CI 1.10-1.62; *P*=.003). There were no significant differences by sex for receiving radiation therapy *(P*=.84) or chemotherapy *(P*=.49). Those older than the median age of 58 years were more likely to receive immunotherapy (OR 1.94, 95% CI 1.60-2.35; *P*<.001). There were no significant differences by age for surgery *(P*=.11), radiation therapy *(P*=.09), or chemotherapy *(P*=.42).

**Table 1:** Treatment modalities, chemotherapies, and immunotherapies in case reports (n=2133).

Mentions				Case report, n (%)
**Treatment modality**				
	**Surgery**			
		Included	693 (32.5)	
		Not included	1440 (67.5)	
	**Radiation therapy**			
		Included	156 (7.3)	
		Not included	1977 (92.7)	
	**Chemotherapy**			
		Included	613 (28.7)	
		Not included	1520 (71.3)	
	**Immunotherapy**			
		Included	597 (28)	
		Not included	1536 (72)	
**Chemotherapy**				
	Dacarbazine		102 (4.8)	
	Cisplatin		88 (4.1)	
	Paclitaxel		62 (2.9)	
	Temozolomide		61 (2.9)	
	Carboplatin		61 (2.9)	
	Nab-paclitaxel		6 (0.3)	
**Immunotherapy**				
	Ipilimumab		341 (16)	
	Nivolumab		272 (12.8)	
	Pembrolizumab		182 (8.5)	
	Atezolizumab		7 (0.3)	
	T-VEC^a^		7 (0.3)	
	Relatlimab		1 (0.05)	

^a^T-VEC: talimogene laherparepvec.

## Discussion

This study explores the demographics represented in melanoma case reports, their treatments, and how treatments vary by demographics. The most common treatment modality was surgery, and the least common treatment modality was radiation therapy. There were significant differences in treatment modalities between sexes, with more male patients receiving immunotherapy and more female patients receiving surgery. Finally, older patients were more likely to receive immunotherapy. Previous work has highlighted the increased stage of melanoma at diagnosis in male patients [3]. Thus, it is plausible that some variations in treatment could be secondary to staging differences. Previous work looking at patients with metastatic melanoma from 2011 to 2015 found that older patients were less likely to receive immunotherapy, despite its greater survival benefit [5]. These differences may stem from practice changes or publication bias. If treatment variations were found to be present in clinical practice, such variations in management by sex could lead to suboptimal patient care and outcomes. Our study was limited in that the use of string-matched case report information may have missed some treatments. Additionally, the PMC-Patients database did not include information on race and ethnicity. Our study highlights the need for more research on treatment variations by demographics in melanoma cases.

## Abbreviations

OR: odds ratio
